# The Potential Role of *Halothiobacillus* spp. in Sulfur Oxidation and Acid Generation in Circum-Neutral Mine Tailings Reservoirs

**DOI:** 10.3389/fmicb.2019.00297

**Published:** 2019-03-08

**Authors:** Kelly Whaley-Martin, Gerdhard L. Jessen, Tara Colenbrander Nelson, Jiro F. Mori, Simon Apte, Chad Jarolimek, Lesley A. Warren

**Affiliations:** ^1^Civil and Mineral Engineering Department, University of Toronto, Toronto, ON, Canada; ^2^Commonwealth Scientific Industry and Research Organization, Clayton, VIC, Australia

**Keywords:** *Halothiobacillus*, sulfur, acidity generation, mining impacted water, acid mine drainage (AMD)

## Abstract

The biogeochemistry of acid mine drainage (AMD) derived from waste rock associated sulfide mineral oxidation is relatively well-characterized and linked to *Acidithiobacillus* spp.. However, little is understood about the microbial communities and sulfur cycling before AMD develops, a key component of its prevention. This study aimed to examine circum-neutral mining impacted water (MIW) communities and its laboratory enrichments for sulfur oxidizing bacteria (SoxBac). MIW *in situ* microbial communities differed in diversity, structure and relative abundance consistent with site specific variations in total aqueous sulfur concentrations (TotS; ~2–17 mM), pH (3.67–7.34), and oxygen (22–93% saturation). However, the sulfur oxidizer, *Halothiobacillus* spp. dominated seven of the nine total SoxBac enrichment communities (~76–100% relative abundance), spanning three of the four mines. The presence and relative abundance of the identified sixteen known and five unclassified *Halothiobacillus* spp. here, were the important clustering determinants across parent MIW and enrichment communities. Further, the presence of *Halothiobacillus* spp. was associated with driving the pH <4 in enrichment experiments, and the combination of specific *Halothiobacillus* spp. in the enrichments affected the observed acid to sulfate ratios indicating differential sulfur cycling. *Halothiobacillus* spp. also dominated the parent communities of the two acidic MIWs providing corroborating evidence for its active role in net acid generation within these waters. These results identify a putative indicator organism specific to mine tailings reservoirs and highlight the need for further study of tailings associated sulfur cycling for better mine management and environmental stewardship.

## Introduction

By 2030, global demand for water is expected to increase to a level 40% above our current supply (World Economic Form, 2014) requiring proactive and novel management approaches to protect the integrity of the global freshwater supply (Richey et al., 2015). Globally, the extractive resource industries collectively use between 7 and 9 billion m^3^ of water every year; representing the second largest industrial user of water after power generation (Barlow, 2013). Mines withdraw water for extraction of desired elements from ore and return the majority of this water to the local environment. Tailings, the combined water-particle waste slurry (~40% solids) generated through ore processing, contains finely ground, highly processed rock particles, and chemical additives added during the metal extraction process. Tailings are stored in retention ponds (variably called tailings dams, dykes, ponds, reservoirs; herein referred to as tailings reservoirs). These reservoirs receive continuous inputs of tailings during operation, as well as additional inputs of mining impacted water (i.e., waste rock run off) from the mine property and catchment inputs. Thus, tailings comprise large volumes of mining impacted water (MIW) that require treatment on site. Without water treatment prior to discharge into receiving environments, significant toxicological impacts to local biota may include exposure to high metal concentrations and acidification (Nordstrom and Alpers, 1999; Sheoran and Sheoran, 2006; Meier et al., 2012). Specifically, ubiquitous sulfur compounds in tailings, derived from sulfidic ore-bearing minerals, are constituents of concern.

The current lack of knowledge on sulfur biogeochemical cycling in MIWs can impede geochemical prediction and management efforts of these waters. The most well known global environmental issue for the mining sector, acid mine drainage (AMD), is characterized by high levels of acidity and metals driven by bacterial oxidation of sulfide minerals (e.g., pyrite, FeS_2_). This process is common for sulfide mineral containing waste rock (displaced during mining activities without being processed), but can also occur within tailings rich in sulphidic minerals (Edwards et al., 1999; Baker and Banfield, 2003; Johnson and Hallberg, 2003; Schippers et al., 2010; Korehi et al., 2014; Méndez-García et al., 2015). AMD results in highly acidic, metal laden waters that contain microbial communities dominated by acidophilic iron and sulfur organisms such as *Acidithiobacillus* and *Leptospirillum* genera (Edwards et al., 1999; Baker and Banfield, 2003; Schippers et al., 2010; Kimura et al., 2011; Kuang et al., 2012). As extraction of sulfide mineral containing ore generates sulfur oxidation intermediate compounds (SOI), most notably thiosulphate (S_2_${\text{O}}_{3}^{2-}$) and other S_n_${\text{O}}_{\text{x}}^{2-}$ compounds (Miranda-trevino et al., 2013), circum-neutral tailings reservoirs will typically contain a variety of aqueous intermediate sulfur substrates. These SOIs can support a more complex and far less understood microbial sulfur cycle than that of waste rock associated AMD (Dockrey et al., 2014; Korehi et al., 2014).

We hypothesize that the subset of the microbial community with the metabolic functionality for sulfur oxidation and acidity production under circum-neutral experimental conditions will be different than organisms traditionally associated with acid mine drainage environments. This work utilized complimentary approaches of geochemical characterization with genetic sequencing to examine microbial communities within circum-neutral mine waters with a focus on the chemoautotrophic sulfur oxidizing organisms. Sulfur-oxidizing bacterial (SoxBac) enrichments of these parent waters were used to delineate these members of the original microbial community and their sulfur oxidation and acid generating capacities. Sulfur oxidizing microorganisms that propagate early stages in acidity generation within circum-neutral tailings ponds may act as useful bioindicators for the onset of AMD conditions and to the best of knowledge have remained largely undefined in these systems.

## Materials and Methods

### Site Descriptions and Sampling Scheme

Nine MIW sites were sampled between September and November of 2017 across four mines: Mine 1 (Cu, Au; Baie Verte, Newfoundland), Mine 2 (Zn, Cu; Snow Lake, Manitoba), Mine 3 (Cu, Ni; Sudbury, Ontario), and Mine 4 (Zn, Cu; Flin Flon, Manitoba; Table 1). In September, each mine's main tailings reservoir was sampled from, and when possible water sampling was also carried out in October (Mine 1) and in November (Mine 3). In addition, an input water source to the tailings reservoir at Mine 3, which combines mine treated water and natural tributaries was collected (September 2017), and a receiving environment site at Mine 4 (September 2017). The receiving environment site at Mine 4 was specially targeted as this local creek has experienced legacy acid mine drainage issues from historical mining activities (communication with mining personnel). Mine 3 has been the focus of past pilot research studies examining the relationships between microbial communities and acidity generation in a tailings reservoir (Bernier and Warren, 2005).

**Table 1:** Physico-and geochemical characterization of the different mining environments used for SoxBac enrichment experiments.

**Field site**	**Depth** **(m)**	**pH**	**Temp (°C)**	**O_**2**_ (%)**	**Total S_***UF***_** **(mmol L^**−1**^)**	**Total S_**0.2μm**_** **(mmolL^**−1**^)**	**S_**2**_**${\text{O}}_{3}^{2-}$ **(mmol L^**−1**^)**	$\text{S}{\text{O}}_{3}^{2-}$ **(mmol L^**−1**^)**	$\text{S}{\text{O}}_{4}^{2-}$ **(mmol L^**−1**^)**	**S_**react**_ (TotS-$\text{S}{\text{O}}_{4}^{2}$) (mmol L^**−1**^)**	**S_**react**_ Proportion (%)**
Mine 1 tailings reservoir (September)	0.5	6.0	–	–	2.3 ± 0.02	2.3 ± 0.01	<0.002	0.006 ± 0.002	2.2 ± 0.4	0.1	4.3
Mine 1 tailings reservoir (October)	0.5	6.96	–	–	2.3 ± 0.01	2.3 ± 0.01	<0.002	–	–	–	–
Mine 2 tailings reservoir (September)	0.5	6.9	9.9	87.7	6.9 ± < 0.01	7.1 ± 0.2	<0.002	0.018 ± 0.001	5.9 ± 0.1	1.0	14
Mine 3 tailings reservoir September	2	4.67	21.4	72.6	10.7 ± 0.2	10.7 ± 0.5	<0.002	0.008 ± < 0.001	6.9 ± 0.2	3.8	36
Mine 3 tailings reservoir (November)	0.5	6.94	11.3	30.6	9.5 ± 0.1	9.4 ± 0.1	–	–	8.3 ± < 0.01	1.2	13
Mine 3 tailings reservoir (November)	10	6.15	11.3	22	7.5 ± 0.04	7.5 ± 0.02	–	–	0.21 ± < 0.01	7.3	97
Mine 3 input water (November)	0.5	3.67	4.6	93.2	10 ± < 0.01	10 ± < 0.01	–	–	6.7 ± 0.1	3.3	33
Mine 4 tailings reservoir (September)	7	7.34	11.1	88.6	17.4 ± 0.9	16.9 ± 0.3	0.31 ± 0.03	0.009 ± < 0.001	11.8 ± 0.7	5.6	32
Mine 4 receiving environment (September)	0.5	4.33	11.0	39.1	14.9 ± 0.3	14.4 ± 0.4	<0.002	0.01 ± < 0.001	10.5 ± 0.6	4.4	30

*Tailings reservoir; Mine 1, 2, 3, and 4; input waters (Mine 3) and receiving environments (Mine 4). Total S; TotS UF, unfiltered and filtered (0.2 μm), S_2_O_3_${\;}_{aq}^{2-}$, SO_3_${\;}_{aq}^{2-}$, S${\text{O}}_{4}^{2-}$ and the reactive S concentration (S_react_; defined as the difference between TotS and sulfate concentrations; values represent average and SD, n = 3)*.

### Physiochemical Characterization and Sampling of *in situ* Mining Wastewater

Each MIW site underwent (1) geochemical characterization (total sulfur (TotS), sulfate, thiosulfate, sulfite, nitrate, and nitrite concentrations), (2) microbial community structure assessment (16S rRNA, Illumina MiSeq), and (3) sulfur oxidizing microbial enrichment (SoxBac) as outlined in Supplemental Material (Figure S1). On site temperature, pH, dissolved oxygen saturation (%), conductivity, and redox as (ORP) were measured with a YSI 600 XLM (Mine 3) and ProDSS water quality meter (Mine 2 and 4). *In situ* physiochemical parameters were not available for Mine 1 tailing reservoirs samples, however pH was measured after arrival to the laboratory. Continued sampling from these sites since the 2017 field campaigns reported in this study confirm that circum-neutral pH (as was measured in the samples upon arrival) is typical of Mine 1's reservoir and therefore considered representative of on-site conditions.

Water samples were collected at Mine 1, 2, and 4 sites and shipped for processing from these remote locations to the University of Toronto, while Mine 3 MIW samples were collected and processed directly on site (Figure S1). Mine 1 and 2 tailing reservoirs are significantly shallower (~1–2 m) than Mine 3 and 4 (~40–10 m, respectively). Water samples from the tailings reservoirs (Mine 3 and 4) were collected using a dedicated Van Dorn sampler as the water columns are significantly deeper, while the remainder of samples were collected using surface grab samplers. Both the VanDorn and surface grab sampler were rinsed with 70% ethanol and rinsed with site water three times prior to sample collection. *In situ* water was collected for geochemical and microbial community assessment from Mine 2 and 4 into 20 liter containers with ethanol-sterilized polyethylene liners (4 mil, polyethylene, 18″ × 24″, Uline S-1379). Liners were fitted into the container and water samples were transferred directly to the liner using either the VanDorn or the surface grab sampler. Once appropriate volumes had been collected (10–20 L), liners were immediately sealed with no headspace. To remain consistent, the same protocol was used to collect water from Mine 3 for microbial community analyses. Mine 1 water samples were collected using ethanol-sterilized plastic containers for sampling and transport of samples. Mine 1 tailing reservoir water was collected by submerging the container until it was overflowing (no headspace).

Due to the remote locations of Mines 1, 2, and 4, water samples were shipped through air transport, which took between 3 and 10 days to arrive at the University of Toronto. Ongoing work is looking at the potential changes to microbial community structure that could occur during that time but all preliminary work to date has supported no significant changes in sulfur speciation occurring during that transport time. This would indirectly support no major activity changes to the sulfur oxidizing portion of the community. Preliminary lab tests have demonstrated the stability of relevant sulfur species (sulfate, thiosulphate, trithionate, and tetrathionate) within these circum-neutral waters for time periods comparable to shipment times (1–10 days) supporting low activity rates of the sulfur oxidizing portions of these microbial communities.

### Geochemical Analyses

Water samples were collected in triplicate (40 mL volume per replicate) for total sulfur (unfiltered water samples; TotS_UF_) and total dissolved sulfur (TotS_0.2um_; MIW filtered through Pall Acrodisc® 25 mm 0.2 μm Supor® membrane filters with polypropylene syringes) into 50 mL Falcon^TM^ tubes pre-spiked with 80 μL of HNO_3_ (Optima grade, Fisher Chemical) and then stored at 4°C until further processing. Analysis was carried out at the Commonwealth Scientific and Industrial Research Organization (CSIRO) through inductively coupled plasma atomic emission spectroscopy (ICP-AES) on a Varian730 ES (Mulgrave, Australia). Certified reference stock solutions (AccuStandard New Haven, CT, USA) were used to prepare sulfur calibration standards in 2% v/v HNO_3_ (limit of detection (LOD) for sulfur was 0.03 mmol L^−1^) and concentrations were determined by measuring intensity at the 181.972 nm sulfur emission line. Background/inter-element interferences were corrected for with the Varian Fast Automated Curve-fitting Technique (FACT).

Thiosulfate (S_2_${\text{O}}_{3}^{2-}$) and sulfite (S${\text{O}}_{3}^{2-}$) analytes were stabilized upon sampling through derivatization with monobromobimane (Rethmeier et al., 1997). Derivatization agents (50 μL of acetonitrile, 50 μL of 50 mmol L^−1^ HEPES (4-(2-hydroxyethyl) piperazine-1-ethanesulfonic acid, ≥99.5%, Sigma), 5 mmol L^−1^ of EDTA buffer (ethylenediaminetetraacetic acid, 99.4–100.6%, Sigma Aldrich; pH = 8.0, adjusted with NaOH) and 10 μL of 48 mmol/L monobromobimane (>97%, Sigma Aldrich) in acetonitrile were added to 50 μL of water sample in 2 mL glass amber vials. The derivatization reaction was carried out in the dark for 30 min, 100 μL of methanesulfonic acid (~100 mmol) (≥99.5%, Sigma Aldrich) was then added to halt the reaction and samples were subsequently kept frozen until analysis. Thiosulphate and sulphite analyses were carried out on a Shimadzu LC-20AD prominence liquid chromatograpy (LC) system coupled to a fluorescence UV/VIS detector on an Alltima^TM^ HP C18 reversed phase column (150 mm × 4.6 mm × 5 μm, Grace^TM^) held at 35°C using an isochratic mobile phase of 65% of 0.25% acetic acid v/v (pH 3.5 adjusted with NaOH) and 35% methanol at 0.5 mL min^−1^ for a total run time of 20 min. The excitation wavelength was set to 380 nm and the emission wavelength 478 nm. The sulfite peak was found to elute at ~5 min and thiosulfate eluted at ~7 min. Calibration curves were prepared using sodium sulphite (Sigma Aldrich, ≥98% purity) and sodium thiosulphate (Sigma Aldrich, 99% purity).

Samples for S${\text{O}}_{4}^{2-}$ and nitrogen species (N${\text{O}}_{2}^{-}$ and N${\text{O}}_{3}^{-}$; see Table S1) analyses were collected with no headspace into 1 liter polypropylene sample bottles rinsed three times with site water and stored at 4°C prior to analysis. Dissolved S${\text{O}}_{4}^{2-}$, N${\text{O}}_{2}^{-}$, and N${\text{O}}_{3}^{-}$ quantification was carried out by spectrophotometry using a HACH DR2800 (HACH Company, Loveland, CO, USA) as described previously in Risacher et al. (2018).

### Microbial Community Structure Characterization (16S rRNA)

#### DNA Extraction and Quantification

Water samples (3 to 7 liters depending on site) were carefully mixed to try and limit settling of dissolved solids and filtered through triplicate 0.2 μm filter towers (Thermo Scientific™ Nalgene™ Rapid-Flow™ Sterile Disposable Filter Units with CN Membrane) as analytical replicates and kept frozen (−20°C) until DNA extraction. DNA extraction was conducted on filters using Qiagen's DNeasy PowerWater DNA Isolation Kit. DNA extracts were sent to the McMaster DNA Sequencing Facility (Hamilton, Ontario) for subsequent analysis. Extracted DNA was quantified by quantitative PCR (qPCR) and its concentrations adjusted for each step of the subsequent molecular protocol.

#### Amplicon and Genome Sequencing

Aliquots of purified DNA were used to amplify region V4 of the 16S rRNA gene by PCR using Illumina adapted primers following Bartram et al. (2011) and standard protocols of the Earth Microbiome Project (Caporaso et al., 2011, 2012). In short, the primers were modified to amplify 515f (GTGYCAGCMGCCGCGGTAA) and 806r (GGACTACNVGGGTWTCTAAT) variable regions of archaeal and bacterial 16S rRNA gene. PCR was performed using 50 ng of template and the PCR mix contained 1U of recombinant Taq DNA Polymerase (Invitrogen™), 1x buffer, 1.5 mmol L^−1^MgCl2, 0.4 mg mL^−1^ BSA, 0.2 mmol L^−1^dNTPs, and 5 pM of each primer. The reaction was carried out at 98°C for 5 min, 35 cycles (98°C) for 30 min, then 30 min (50°C), and 30 min (72°C), with a final extension of 72°C for 10 min. PCR products were checked by electrophoresis and sent for sequencing. All amplicons were normalized to 1.25 ng μL^−1^ using the SequalPrep normalization kit (ThermoFisher#A1051001) and sequenced using the Illumina MiSeq platform. The percentage of reads that passed the quality control parameter “Q30” for each of the triplicates runs were 85, 89, and 90%, respectively. Bimera checking was performed on the data carried out through DADA2 (version 1.6.0) and 3–5% of reads that were detected as bimeras and excluded.

#### Bioinformatics and Statistical Analyses

Raw sequences were filtered and trimmed using *Cutadapt* with a minimum quality score of 30 and a minimum read length of 100 bp (Martin, 2011). Sequence variants were then resolved using DADA2 version 1.6.0 (Callahan et al., 2016) and its reads filtered and trimmed based on the quality for each Illumina run separately. Sequence variant tables were merged to combine all information from separate Illumina runs, while bimeras were removed along with sequences identified as chloroplasts or mitochondria. Taxonomy was assigned using the SILVA database version 132 and microbial function was inferred from cultured representatives of the identified sequences. Sequences obtained in this study were deposited at NCBI under the bioproject accession number PRJNA510600.

Alpha and beta diversity was calculated from the relative abundance of sequence variants, whereas patterns in community composition were explored using non-metric multidimensional scaling (NMDS) and hierarchical clustering, based on Bray–Curtis dissimilarities. Analysis of similarity (ANOSIM) was used to test differences between groups (i.e., mines and enrichments) and *p*-values corrected using Bonferroni's correction. All statistical analysis were conducted following Buttigieg and Ramette (2014) and Ramette (2007) and using vegan R scripts (Oksanen et al., 2010) (version 3.4.4; www.R-project.org).

Phylogenetic trees were created using the amplicon sequences (~264 bp) based on maximum-likelihood using MEGA (v7.0.26) (Kumar et al., 2016). As a reference, the 16S rRNA gene sequences of different known sulfur oxidizing microorganisms were selected from the databases of SILVA (version 132) and National Center for Biotechnology Information (NCBI). The nucleic acid sequences selected were aligned using ClustalW in MEGA and the phylogenetic tree was created using Tamura-Nei model with 1000 bootstrap iterations.

### Sulfur Oxidizing Bacterial (SoxBac) Enrichments From Mining Wastewaters

Water samples for enrichments were taken directly on site (Mine 3) or from shipped water (Mine 1, 2, and 4; Figure S1) in 90 mL sterile containers with no headspace and stored at 4°C until enrichment for SoxBac was initiated (within 2–5 days of collection). The indigenous microbial communities from the active mine wastewater systems were enriched for sulfur oxidizing bacteria (SoxBac) in a neutral pH medium (pH~7) that contained thiosulphate (S_2_${\text{O}}_{3}^{2-}$) as sulfur substrate. Thiosulphate was chosen as a suitable sulfur substrate as it is known to be an important intermediate in microbial sulfur cycling and is an abundant and ubiquitous SOI generated during metal extraction from sulfide ores (Warren et al., 2008; Shi et al., 2011; Veith et al., 2012; Miranda-trevino et al., 2013; Liu et al., 2014). Thiosulphate is also monitored most frequently by industrial monitoring programs (Whaley-Martin et al. in preparation). Methodology for growth of sulfur oxidizing bacteria was based on those described elsewhere in detail (Bernier and Warren, 2005). Briefly, three cycles were employed to generate an active SoxBac enrichment from each of the nine parent water samples. Preliminary experiments comparing enrichments grown for 3, 4 and 5 cycles found that rates of acid production were relatively stable in the third cycle, indicating that SoxBac activity was sufficient.

In the first cycle, a 1:1 ratio of mining water to the neutral pH media (described in SI) was prepared under sterile conditions in 150 mL autoclaved flasks in a Biological Safety Cabinet (BSC). SoxBac enrichments samples were then incubated under dark conditions at room temperatures (~comparable to *in situ* MIW temperatures) until they reached a pH <5. Monitoring of pH changes (used as a proxy for microbial sulfur metabolic activity) was carried out with a sterile probe (immersed in 70% ethanol for a minimum of 2 min and rinsed with sterile (0.2 μm filtered) MilliQ water (Bernier and Warren, 2005). The second and third successive cycles of SoxBac enrichments were created by sterile pipetting 50 mL of cycle one or two SoxBac enrichment water to an autoclave flask with 100 mL of new media (1:2 ratio of parent water to media). The pH was adjusted to ~7 with dilute sterile (0.2 μm filtered) NaOH/HCL solution and enrichments were monitored under the same conditions as cycle 1 and then halted once the pH had decreased <5 (Cycle 3: 3 to 6 days, Table 2). For cycle 3, both initial and final enrichment waters were sampled for geochemistry (pH, S${\;}_{2}{\text{O}}_{3}^{2-}$, S${\text{O}}_{3}^{2-}$, S${\text{O}}_{4}^{2-}$, and TotS_UF and 0.2μ*m*_ determination) and final waters for enrichment consortia characterization (16S rRNA) following the protocols outlined above. Control samples consisting of media and sterilized mine water for each parent sample were also monitored for pH and S${\text{O}}_{4}^{2-}$, S${\;}_{2}{\text{O}}_{3}^{2-}$, and S${\text{O}}_{3}^{2-}$ concentrations to determine any abiotic SOI alteration that may have occurred. Control samples showed pH changes only fluctuated around circum neutral conditions (~7–8) and SOI species changes were < LOD.

**Table 2:** Initial and final sulfur speciation and pH in sulfur oxidizing microbial enrichments on their 3rd cycle grown from parent communities from the nine MIW samples.

	**Cycle duration** **(days)**	**pH**		**S-S**_**2**_${\text{O}}_{3}^{2-}$ **(mmol L^**−1**^)**			**S-**$\text{S}{\text{O}}_{3}^{2-}$ **(mmol L^**−1**^)**			**S-**$\text{S}{\text{O}}_{4}^{2-}$ **(mmol L^**−1**^)**			**S-unresolved** **(mmol L**^****−1****^**)**		
		***I***	***F***	***I***	***F***	**Δ%**	***I***	***F***	**Δ%**	***I***	***F***	**Δ %**	***I***	***F***	**Δ %**
E Mine 1	5	7.1	6.1	19.9 ± 1.4	17.9 ± 0.2	−10	0.048 ± 0.01	0.39 ± 0.03	700	9.2 ± 0.6	12.4 ± 0.6	+34	26.9 ± 2.0	25.3 ± 2.0	−6
September															
Tailings Reservoir															
0.5 m															
E Mine 1	3	7.0	6.7	19.8 ± 1.1	20.0 ± 0.5	+1	0.074 ± 0.046	0.03 ± 0.002	−59	4.3 ± 0.3	4.6 ± 0.7	+7	31.8 ± 2.7	31.4 ± 2.7	−1
October															
Tailings Reservoir															
0.5 m															
E Mine 3	6	7.0	3.1	18.8 ± 1.4	0.1 ± 0.02	−99	0.024 ± 0.003	<0.01	−58	11.2 ± 0.6	14.4 ± 1.0	+29	30.0 ± 1.1	45.5 ± 1.1	+52
November															
Tailings Reservoir															
0.5 m															
E Mine 3	6	7.0	3.0	19 ± 0.4	0.2 ± 0.05	−98	0.039 ± 0.007	<0.01	−74	12.0 ± 0.2	15.0 ± 0.8	+25	28.7 ± 1.1	44.8 ± 0.2	+156
November															
Tailings Reservoir															
10 m															
E Mine 2	5	7.1	3.0	20.0 ± 1.7	0.1 ± 0.02	−99.5	0.03 ± 0.02	<0.01	−66	12.8 ± 0.1	23.3 ± 0.4	+82	22.3 ± 2.1	31.6 ± 2.1	+142
November															
Input Water															
0.5 m															
E Mine 4	5	7.0	3.1	20.3 ± 0.9	0.1 ± 0.01	−99	0.07 ± 0.05	<0.01	−86	13.5 ± 0.3	22.5 ± 0.6	+67	25.1 ± 2.0	36.4 ± 2.0	+45
September															
Tailings Reservoir															
7 m															
E Mine 4	5	7.0	3.9	19.6 ± 0.7	0.2 ± 0.04	−99	0.07 ± 0.04	<0.01	−86	11.3 ± 0.03	21.1 ± 0.6	+87	26 ±*NA*	35.7 ±*NA*	+37
September															
Receiving Environment															
0.5 m															
E Mine 3	6	7.1	3.0	20 ± 1.7	0.1 ± 0.02	−99.5	0.04 ± 0.01	<0.01	−75	10.1 ± 0.3	17.5 ± 1.1	+73	30.7 ± 0.7	43.4 ± 0.7	+41
September															
Tailings Reservoir															
0.5 m															
E Mine 3	7	7.0	3.1	19.7 ± 0.3	0.1 ± 0.05	−99.5	0.059 ± 0.05	<0.01	−83	8.3 ± 0.6	21.51 ± 0.6	+159	19 ±*NA*	25.3 ±*NA*	+33
September															
Tailings Reservoir															
2 m															

*Unresolved sulfur (S-unresolved) was determined by S mass balance subtracting the concentrations of all measured sulfur species from TotS_UF_ concentrations*.

## Results

### Sulfur Geochemistry of Parent MIW and Enrichments

Five of the six tailings reservoir MIW samples were circum-neutral (pH 6–7.3), while the Mine 3 September tailings reservoir 2 m depth (pH of 4.7), and input water sample (3.7) and Mine 4 receiving environment sample were acidic (4.3; Table 1). Concentrations of TotS_UF_ ranged from 2.3 (Mine 1) to 17.5 (Mine 4) mmol L^−1^ with similar ranges seen in the 0.2 μm filtered and TotS fraction of waters (2.3–16.9 mmol L^−1^) indicating minimal particulate S species within these samples (Table 1). S${\text{O}}_{4}^{2-}$ concentrations ranged from 2.2 mmol L^−1^ (Mine 1 tailings reservoir) to 11.8 mmol L^−1^ (Mine 4 tailings reservoir) across all samples (Table 1). Sulfur mass balance identified that a pool of reactive sulfur (available for sulfur oxidation; S_react_: [TotS]—[S${\text{O}}_{4}^{2-}$]) ranged from 1% (at Mine 3 Tailings Pond (0.5 m) in September) to 97% (10 m at Mine 3 in November; Table 1). Thiosulphate concentrations were less than the detection limit for all samples except for the Mine 4 tailings reservoir MIW sample, where a concentration of 0.31 mmol L^−1^ was detected. Sulfite concentrations were low but detectable, varying between 6 and 18 μmol L^−1^ across samples (Table 1). Thus, most of these waters contained 1–7 mmol L^−1^ of intermediate oxidation state sulfur (in other SOI compounds than thiosulphate or sulphite) that can support microbial sulfur oxidative metabolism.

A SoxBac enrichment was successfully generated from the MIW collected at all nine sites consistent with the detectable presence of sulfur at mmol L^−1^ concentrations within these waters, even at Mine 1, which had the lowest TotS of 2.3 mmol L^−1^ and the lowest S_react_ of 100 μmol L^−1^ (Table 1). Each SoxBac enrichment began with ~20 mmol L^−1^ S-S_2_${\text{O}}_{3}^{2-}$ ending in depleted thiosulphate to concentrations approaching the analytical limits of detection (<0.001 mM), with the exception of those grown from Mine 1 tailings reservoir water (Table 2).

This result was accompanied by high and comparable acidity generation rates (consistent with sulfur oxidation pathways) where pH decreased from circum-neutral conditions (pH ~7) to ~3 in 5–7 days for Mine 2, 3, and 4 MIW enrichments. The SoxBac enrichment grown from September Mine 1 tailings reservoir was only able to decrease pH one unit (6.1) in 5 days but this was the only enrichment experiment where sulfite production was detected (Table 2). Interestingly, the net concentration of sulfate produced was similar between Mine 1 tailing reservoir Sept SoxBac enrichment and Mine 3 Nov 10 m tailing reservoir (3.2 and 3 mmol L^−1^ respectively, Table 2). However, the Mine 3 enrichment pH dropped to 3.03 by the end of the enrichment cycle, generating 0.93 mmol L^−1^ more of H^+^ than the Mine 1 SoxBac enrichment (Table 2). Further, this Mine 3 SoxBac enrichment converted 18.6 mmol L^−1^ of S in S_2_${\text{O}}_{3}^{2-}$, compared to only 2 mmol L^−1^ S-S_2_${\text{O}}_{3}^{2-}$ converted in Mine 1 September tailings reservoir SoxBac enrichment. The ratio of net concentration change of sulfate to thiosulfate (*t* = initial vs. *t* = final) and the net production of [H^+^] from initial to final (Figure S2) suggest that Mine 1 SoxBac enrichments should be the most dissimilar to the other enrichment communities.

#### Microbial Community Composition in Circum-Neutral Mine Tailings Reservoirs and SoxBac Enrichments

**Figure 1:**
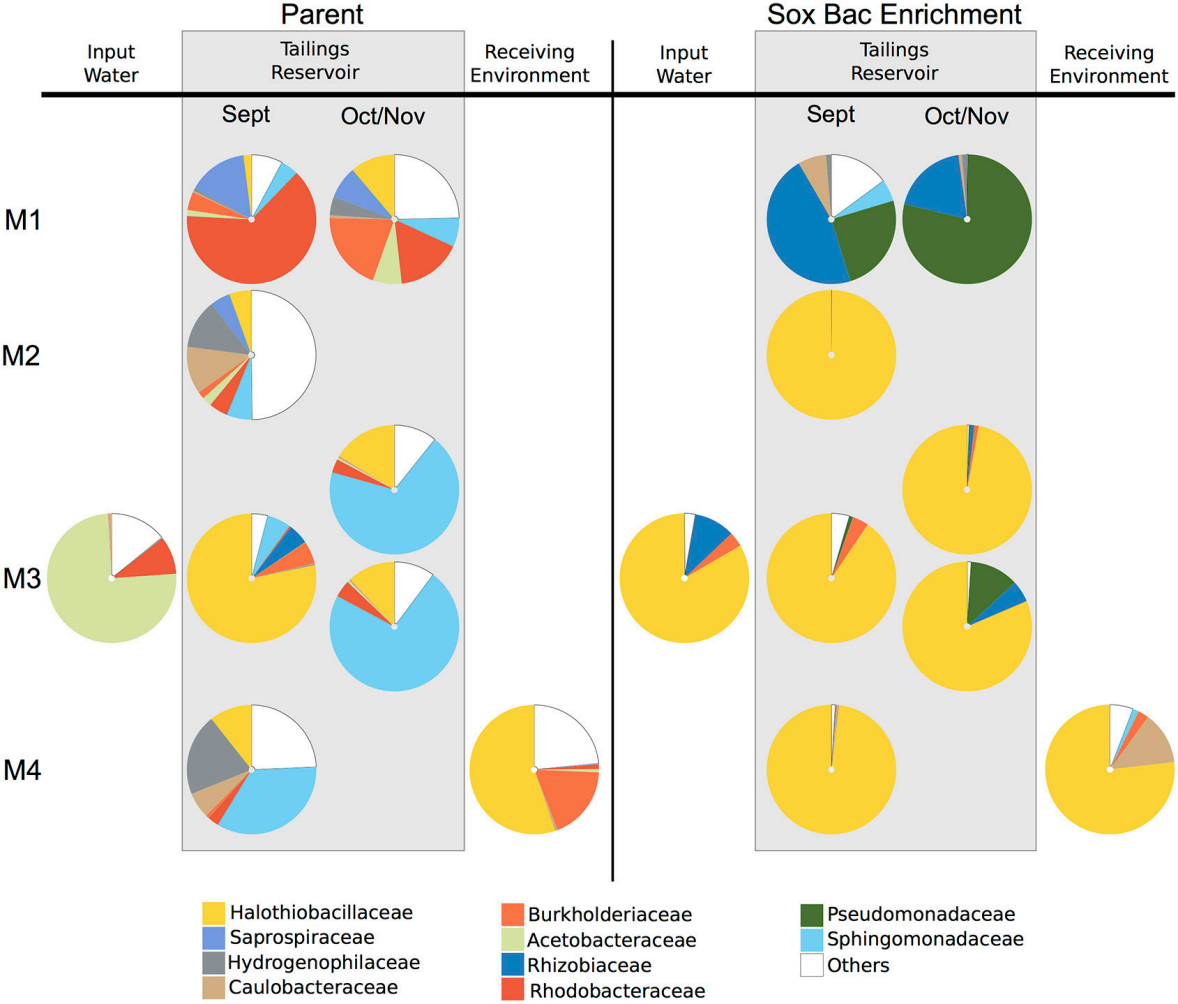
Relative sequence abundance of bacterial families across mine environments. Sequences were obtained by Illumina sequencing of 16S rRNA gene amplicons **(A)** in parent communities (*n* = 9) of mining wastewater at Mine 1 (M1) [Tailings reservoirs (0.5 m) in September and October], Mine 2 (M2) [Tailings reservoir (0.5 m) in September], Mine 3 (M3) [Tailings reservoir (2 m) in September, Input water, and Tailings Reservoir (0.5 m and 10 m) in November], and Mine 4 (M4) [Tailings reservoir (7 m) and Receiving Environment in September] and **(B)** the communities grown through sulfur oxidizing bacterial (SoxBac) enrichment experiments (*n* = 9). “Others” is defined as organisms that had lower relative abundance than the 10 bacterial families with the highest proportions across all samples.

A total of 995,163 and 1,378,115 sequence reads were recovered from the parent MIW communities and their associated SoxBac enrichments, respectively (Table S2). The parent MIW communities showed high diversity across mines and seasonal samples (Figure 1), with the families *Rhodobacteraceae* dominating at Mine 1 tailings reservoir, *Sphingomonadaceae* dominating at Mine 3 (November), and Mine 4 tailings reservoir, *Halothiobacillaceae* dominating the Mine 3 tailings reservoir in September, and “*Others”* dominating at the Mine 2 tailings reservoir. “Others” is defined as organisms that had lower relative abundance than the 10 families with the highest proportions across all samples. The acidic input sample to Mine 3 and receiving environment sample for Mine 4, showed minimal similarity to their respective associated tailings reservoir microbial community structure, with *Acetobacteraceae* dominating the Mine 3 input and *Halothiobacillaceae* dominating the Mine 4 receiving environment (Figure 1).

The microbial community in the Mine 1 tailings reservoir in September (0.5 m depth) was dominated by the family *Rhodobacteraceae* (59%) and the family *Halothiobacillaceae* present at 2% (Figure 1). In October, a shift in the microbial community within this tailings reservoir was observed with *Rhodobacteraceae* decreasing in abundance (16%) and the family *Halothiobacillaceae* increasing (11%). The community of Mine 2 tailings reservoir (1 m depth) in September 2017 had the most even distribution in terms of relative abundance with the families *Halothiobacillaceae* (5%), *Saprospiraceae* (5%), *Hydrogenophilaceae* (12%), *Caulobacteraceae* (11%) and *Sphingomonadaceae* (6%) comprising 39% of the overall distribution. In Mine 3 tailings reservoir (sampled in September from 0.5 m), *Halothiobacillaceae* dominated the distribution at ~78% with *Sphingomonadaceae (*~*6%)*. In Mine 3 tailings reservoir (sampled in November 2017 during a lake turnover event), the two depths (0.5–10 m) had highly similar distributions, dominated by *Sphingomonadales* (68–72%) followed by the family *Halothiobacillaceae* (16–12%, respectively) (Figure 1). The acidic input water of Mine 3 was dominated by *Acetobacteraceae* (71%) and *Rhodobacteraceae* (9%), while the family *Halothiobacillaceae present* at <1%. In the Mine 4 tailings reservoir, *Sphingomonadaceae* (34%), *Hydrogenophilaceae* (20%), and the family *Halothiobacillaceae* (10%) comprised 70% of the microbial community relative abundance distribution (Figure 1). The receiving environment for Mine 4 was dominated by *Halothiobacillaceae* (53%) and *Burkholderiaceae* (18%).

Contrasting the divergent communities observed in the parent MIW samples, the microbial communities in Mines 2–4 SoxBac enrichments were all significantly dominated by the family *Halothiobacillaceae* (~76–100%; Figure 1). These were comparable proportions to those observed in the acidic receiving environment for Mine 4 and the one acidic tailings reservoir MIW sample for Mine 3 (September 2 m depth) but contrasting the parent water community observed for the acidic input water to Mine 3 in September which was the only sample dominated by *Acetobacteraceae*. In contrast, and as suggested by the different sulfur geochemical results obtained for the Mine 1 parent MIW samples (Table 2, Figure 1; Figure S2), Mine 1 SoxBac enrichments diverged from the other enrichments, dominated by *Pseudomonadaceae* (79–25%) and *Rhizobiaceae* for both September and October samples. Control samples were sequenced and re-confirmed the sulfur geochemical and pH characterization results that analytical contamination did not occur as *Halothiobacillus* (the dominating member of all other enrichments) was not present in the control samples.

**Figure 2:**
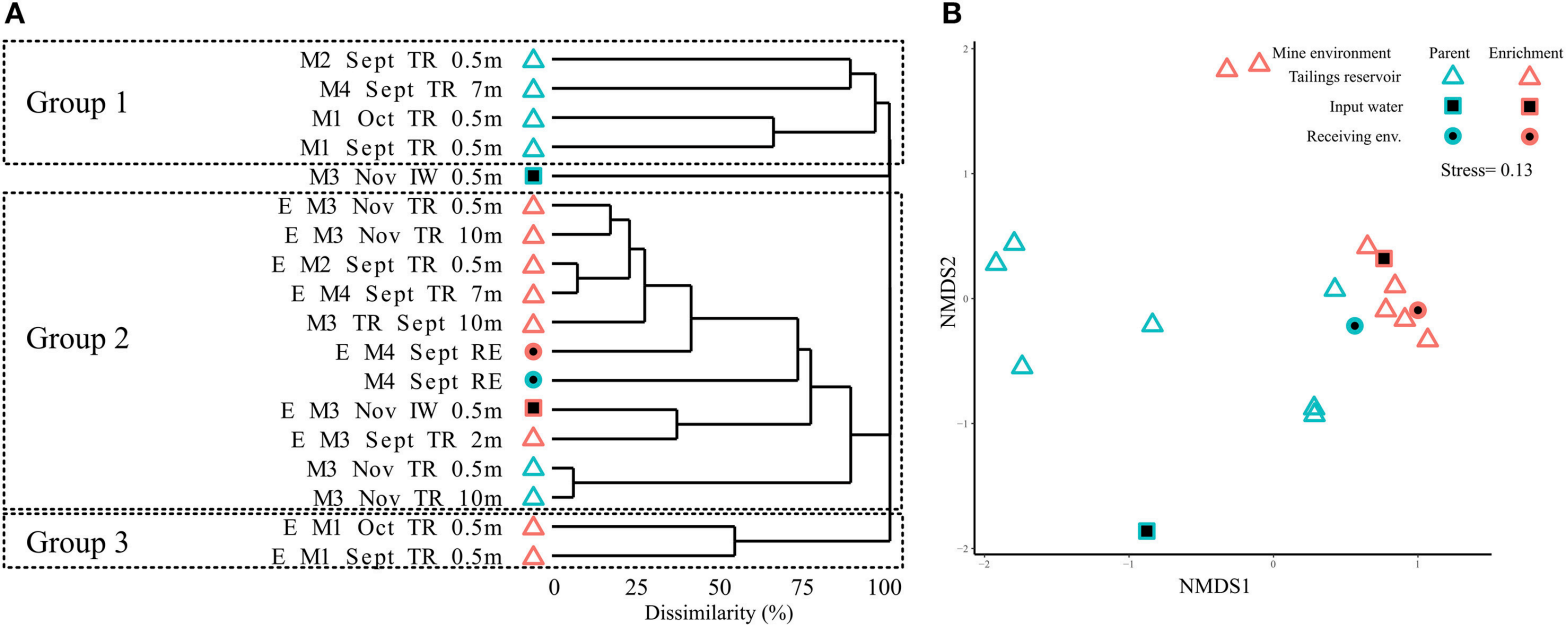
Microbial community structure for different mine environments. **(A)** Bray-Curtis dissimilarity based hierarchical clustering of samples at sequence variants level (obtained by obtained by Illumina sequencing of 16S rRNA gene amplicons). M, mine; E, enrichment; TR, tailings reservoir; IW, input water; RE, receiving environment. Sampling depth is surface water unless depicted (m). **(B)** Non-metric multidimensional scaling (NMDS) plot of samples at sequence variants level (based on Bray-Curtis dissimilarity from Illumina sequencing of 16S rRNA gene amplicons), depicting different mine environments and differences between microbial community structure of enrichments and mines (ANOSIM; *R* = 0.42; *P* < 0.001).

**Figure 3:**
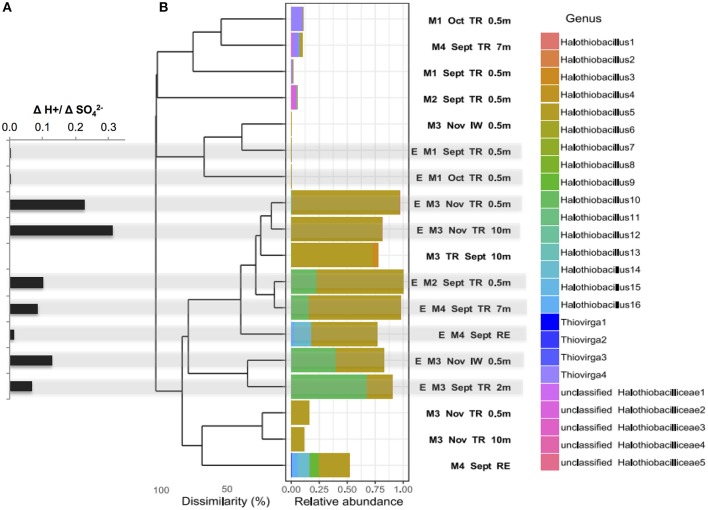
**(A)** Net acid generation relative to net sulfate generated ([H^+^:[S${\text{O}}_{4}^{2-}$]) (bold stars) for each SoxBac enrichment and **(B)** Bray-Curtis dissimilarity based hierarchical clustering of samples for all the single absolute sequences clustered at genus level for the family *Halothiobacillaceae*. Sequences were obtained by Illumina sequencing of 16S rRNA gene amplicons for the different mines, mining environments and its enrichments. *Halothiobacillaceae* relative abundance is calculated per sample and relative to the whole microbial community. Gray shading indicates the ΔH^+^:ΔS${\text{O}}_{4}^{2}$- ratios that correspond to each enrichment SoxBac community.

Clustering of MIW parent communities and SoxBac enrichments revealed three distinct groups that did not cluster either by parent MIW or SoxBac enrichments, or by Mine (Figure 2A) with significant differences observed (Figure 2B, ANOSIM; *R* = 0.42; *P* < 0.001). Rather, the genus *Halothiobacillus* spp. was the dominant OTU driving the observed clustering. Mine 3 input water's microbial community differed from all other sites and did not fall within any of the three groups. The first group of microbial communities consisted of Mine 1, 2, and 4 tailings reservoirs. This group generally had higher sequence richness 286 ± 140 (Table S1) and lower relative abundance of *Halothiobacillus* spp. (Table S4). The second group comprised of Mine 2, 3, and 4 SoxBac enrichments, Mine 3 tailings reservoir samples (September and November samples, 0.5, 2, and 10 m), and the Mine 4 receiving environment sample. This group had a higher relative abundance of *Halothiobacillus* spp. and typically a lower overall richness averaging 67 ± 60 (with the exception of the Mine 4 receiving environment that had the highest overall richness (702, Table S2) of all samples examined in this study). The third group was comprised exclusively of the two Mine 1 SoxBac enrichments. This group was distinct in composition as the genus *Halothiobacillus* spp. was <0.05% of the overall composition, despite being grown with the same reactive sulfur substrate (thiosulphate) and under the same conditions as all the other SoxBac enrichments. Notably, the parent community in Mine 1 tailing reservoir also possessed the family *Halothiobacillaceae* in September and October (Figure 3A).

The SoxBac enrichments grown from Mine 2, 3, and 4 samples (tailings reservoir, input, and receiving environment water samples), which all contained *Halothiobacillus* spp., showed a relatively higher H^+^: S${\text{O}}_{4}^{2-}$ production (Figure 3A; Table 2) compared to Mine 1 SoxBac enrichments. The bacterium affiliated with family *Halothiobacillaceae*, the candidate key sulfur oxidizers detected here from *in situ* MIW and the SoxBac enrichments, was comprised of three genera: *Halothiobacillus, Thiovirga* and an unclassified genus, respectively (Figure 3B). Mines 2, 3, and 4 *in situ* tailings reservoir waters were dominated by the genus *Halothiobacillus* and show the highest relative generation of acid to sulfate concentrations (Figures 3A,B)*. Sixteen* different sequences within the Family *Halothiobacillaceae* were found at the genus level numbered *Halothiobacillus* 1–16 (Figure 3B) and enrichments were dominated by *Halothiobacillus 5, 10*, and *14*. The highest H^+^/S${\text{O}}_{4}^{2-}$ (0.23–0.31) ratios were present in the enrichments where *Halothiobacillaceae* 5's relative abundance was >75% grown from Mine 3 tailings reservoir at 0.5 and 10 m in November (Figure 3). However, ratios were lower when *Halothiobacillaceae* 10 and 14 combined made up a proportion of the overall community ranging from 10 to 67% (Figure 3, Table S4).

**Figure 4:**
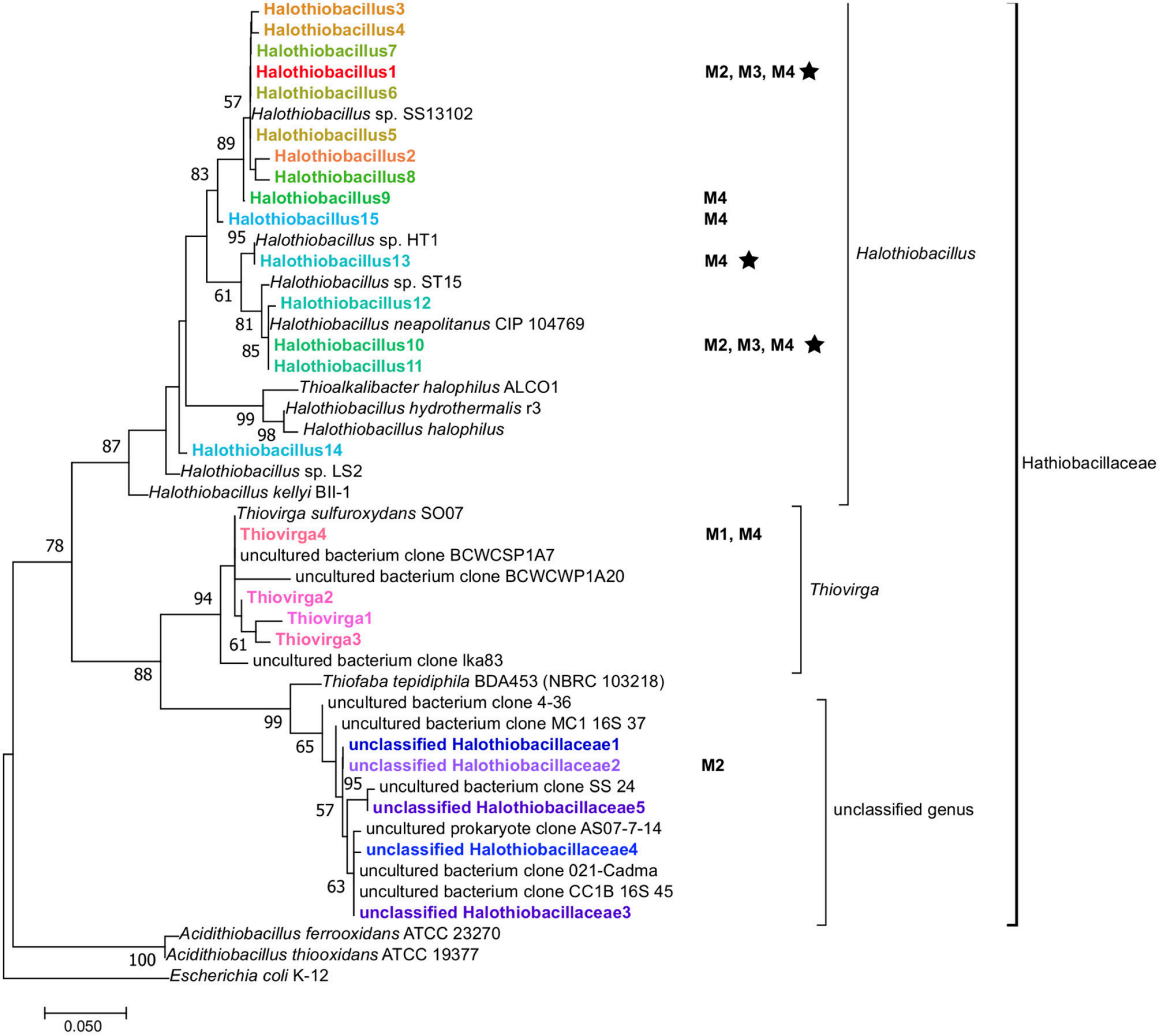
Maximum-likelihood phylogenetic tree of 16S rRNA gene amplicons (~264 bp) of the family *Halothiobacillaceae* detected from different mine parent water samples (M1–4) and SoxBac enrichments (in bold) showing the affiliation of sequences of the family *Halothiobacillaceae*. Reference genes of known *Halothiobacillaceae* (isolates and uncultured clones), and *Acidithiobacillus* spp. in the databases of SILVA and NCBI are also shown and the tree was rooted with *E. coli* K-12. The tree was created with 1,000 bootstrap iteration and the values below 50% are not reported. The scale bar represents 5% sequence divergence. Some amplicons are shown with their origins (Mines 1 to 4 (“M1–M4”) when their relative abundances in whole microbial 16S rRNA gene community are more than 1%. The star symbol indicates organisms that were cultured in the SoxBac enrichments from the parent mining waters.

Mine 1 SoxBac enrichment communities were the only samples that did not contain high abundances of *Halothiobacillus* spp. (<0.03 %). These enrichments were grown from parent waters in which *Thiovirga* was the dominant member of the *Halthiobacillicaea* family (Figures 3, 4). Mine 3 waters also contained *Thiovirga*, but at a considerably lower proportion than *Halothiobacillus* (Figure 3). Four orders of magnitude lower acid generation rates (~10^−7^ mol H^+^ per day) were observed from the Mine 1 tailings reservoir SoxBac enrichments accompanied by a considerably smaller decrease in [S_2_${\text{O}}_{3}^{2-}$] throughout the final generation (Table 2). As mentioned, the production of sulphite from thiosulphate was only observed to occur in the Mine 1 SoxBac enrichments, which might support sulphite as an oxidative intermediate in the sulfur pathways catalyzed by the SoxBac enrichments from Mine 1.

## Discussion

### Differences Between Microbial Communities in Circum-Neutral MIWs and Acid Mine Drainage Environments

The biogeochemical research in mining environments to date has been heavily focused on AMD sites (e.g., waste rock containing sulfide minerals) while the organisms governing the geochemistry in circum-neutral tailing reservoirs remains poorly understood. In this study, there was a consistent absence of the extreme acidophilic iron and sulfur organisms typical of AMD environments (Edwards et al., 1999; Baker and Banfield, 2003; Schippers et al., 2010; Kimura et al., 2011; Kuang et al., 2012). These results are not surprising given the circum-neutral pH values of many of the tailings reservoir MIW samples investigated here. However, even in the three acidic MIW samples *Acidithiobacillus* or *Leptospirillum* spp. were not detected (Figure 1). Results further show that the parent mine tailing reservoir MIWs (Mine 1, 2, 3, and 4), input water (Mine 3) and receiving environment (Mine 4; Table 1) samples were comprised of diverse and distinct microbial communities (Figure 1), consistent with the wide range of geochemical conditions present across the parent waters (Table 1). *Sphingomonadaceae* were consistently present in the microbial communities of the four tailings reservoirs examined in this study, with relative abundances ranging from 4 to 72%. *Sphingomonadaceae* featured prominently in both Mine 3 (September) and Mine 4 (November) tailings reservoir communities, which were both circum-neutral and exhibited similar temperature (~10°C) and oxygen saturation values >50% (Table 1). *Sphingmonadaceae* have been identified as organic biodegraders (White et al., 1996) with a high metal tolerance (Liu et al., 2017). In the Mine 1 tailings reservoir, also circum-neutral, but exhibiting the lowest Total S concentration of 2.3 mmol L^−1^ (Table 1) and the highest nitrate concentration (Table S1), the microbial communities were dominated by *Rhodobacteriaceae* (September) along with *Burholderiaceae* (October). Both of these families have been associated with sulfur, carbon and nitrogen biogeochemical cycling (i.e., Ziga et al., 2010; Pujalte et al., 2014).

### Sulfur Oxidizing *Halothiobacillus* spp. as an Early-Indicator of Acid Mine Drainage

*Halothiobacillus* spp. emerged as an important sulfur oxidizing bacterium present in three of the four mines, capable of driving circum-neutral pH to acidic conditions, whose presence and relative abundance was the most important factor associated with the observed clustering of parent MIW and enrichment communities (Figures 1–3). *Halothiobacillus* spp. is a motile chemolithoautotrophic sulfur oxidizing Gammaproteobacterium capable of oxidizing sulfide, elemental sulfur, thiosulfate and tetrathionate to sulfur, sulfite, polythionates, and sulfate (Kelly and Wood, 2000; Sievert et al., 2000; Shi et al., 2011). The acidophilic organisms that dominate AMD environments are likely end-members of a microbial succession where circum-neutral (or alkaline) conditions are biologically transformed through oxidative processes to acidic conditions. In this scenario, these organisms colonize these waters subsequent to mesophillic or moderate acidophilic microbial communities (Korehi et al., 2014). Liu et al. (2014) reported a relationship between microbial communities in mine tailing cores and overall pH, which in that study ranged from circum-neutral to pH 2–3. Specifically, the class Gammaproteobacteria was implicated in possessing a relationship to pH conditions within the mine tailings (Liu et al., 2014). This is consistent with the Gammaproteobacterium, *Halothiobacillus* dominating SoxBac enrichments from Mine 2–4 and two *in situ* MIWs where pH was measured to be ~4 observed in this study (Figure 2, Table 2). As mentioned, the genus *Halothiobacillus* was more prominent in two of the three acidic parent waters [Mine 3 tailings reservoir (September) and Mine 4 receiving environment (Table 1)], while the family *Acetobacteraceae* were dominant in the acidic input water to Mine 3. Unlike *Halothiobacillus, Acetobacteraceae* has not been largely associated with sulfur cycling and instead is associated with carbon cycling and propagating acidic environments (White et al., 1996). As the input water at Mine 3 contains a combination of tailings water and natural tributaries, there maybe a flux of organics from natural sources additionally into this sulfur containing water that supports both heterotrophic and acid producing roles of the community.

**Figure 5:**
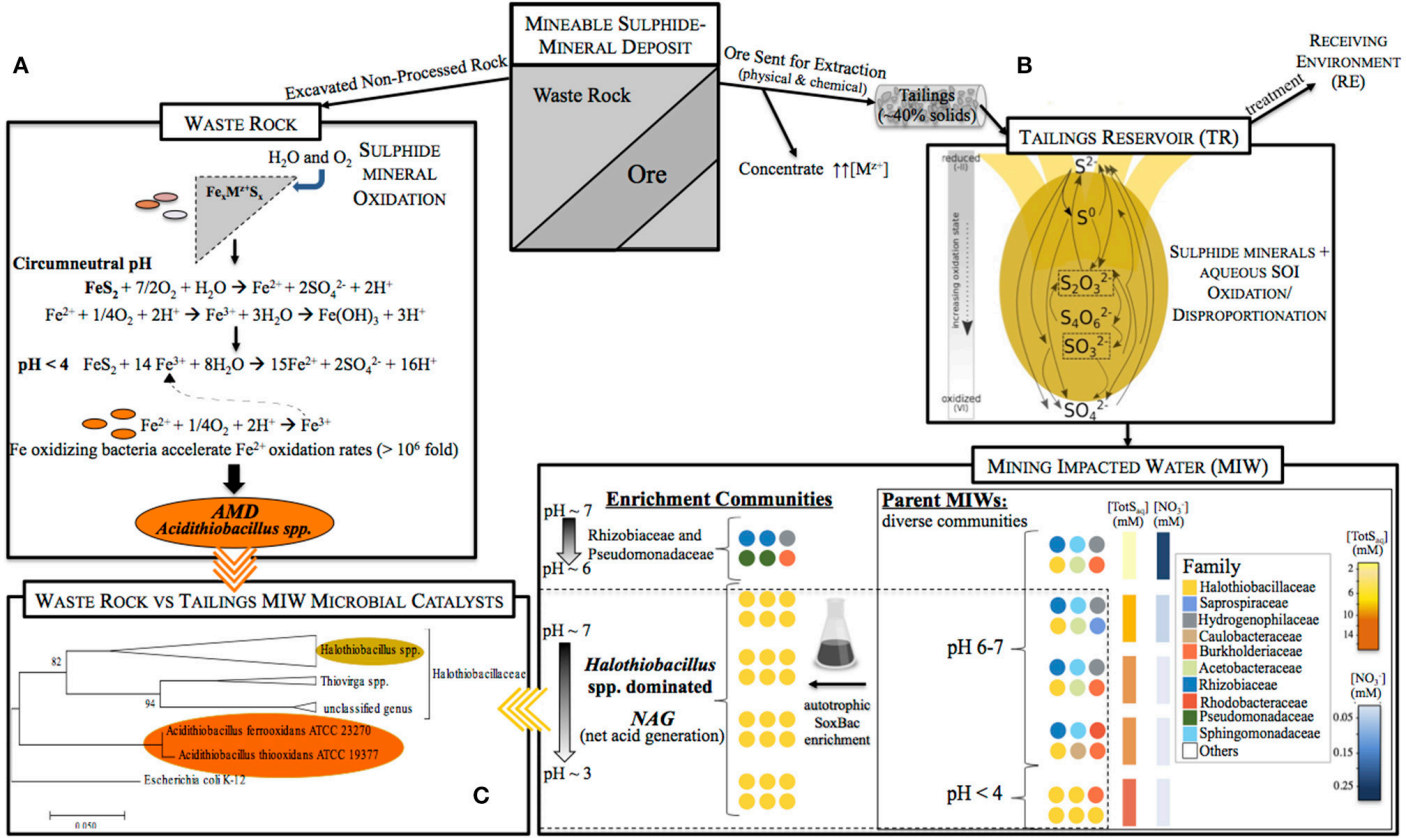
Mining of FeS_x_ minerals creates sulfide rich waste rock **(A)**, as well as post extraction sulfide rich tailings managed in tailings reservoirs **(B)**. Results of this study show that the sulfur geochemical reactions that occur, as well as the important microbial players associated with either AMD **(A)** or NAG **(B)** conditions in these two contexts differ. Waste rock contexts are dominated by Fe and S oxidizing acidophiles associated with the iron and sulfur rich minerals hosted in these materials **(A)**. In contrast, tailings reservoirs exhibit far greater geochemical diversity and sulfur compound complexity **(B)**. Results here identify that parent microbial communities differ across tailings reservoir MIW samples consistent with variable geochemistry **(B)**. However, SoxBac enrichments experiments identify the genus *Halothiobacillus* spp. as an important driver of NAG generation that appears widespread within these tailings reservoir MIWs. **(C)** Phylogenetic relationship/distance between *Halothiobacillus* spp. and *Acidothiobacillus ferridoxans* (a well-known organism associated with AMD conditions) indicate that these are not closely related organisms indicating that AMD based understanding of microbial sulfur cycling will not accurately reflect NAG generation within tailings reservoirs.

Although *Halothiobacillus* has been shown to be a rare group within AMD environments (Schippers et al., 2010; Yang et al., 2014), our results show that this group can account for more than 70% of the microbial community in SoxBac enrichments derived from tailings associated MIWs (**Figure 5**). The parent waters where *Halothiobacillus* were detected here (Mines 2–4), contained considerably higher total aqueous sulfur concentrations (7–17 mM) than Mine 1 (~2 mM) and concentrations of nitrate two magnitudes lower than Mine 1 (Table 1; Table S1) discussed further below. While optimal growth conditions for *Halothiobacillus* occur under circum-neutral conditions, supporting a classification of “moderate acidophile” (Kelly and Wood, 2000; Sievert et al., 2000; Schippers et al., 2010; Marescotti et al., 2012; Yang et al., 2014), Sievert et al. (2000) reported that laboratory *Halothiobacillus* incubations under neutral conditions were driven as low as pH~3 through sulfur oxidation. Moreover, Shi et al. (2011) isolated an unspecified strain of *Halothiobacillus* from a lead-contaminated soil and reported a high metal tolerance, consistent with the mining environments assessed here. Thus, *Halothiobacillus* appears to be highly adapted to metal mine environments due to its ability to tolerate elevated metal conditions (Table S3) and oxidize an array of reduced sulfur compounds and phylogenetic diversity (Figures 3 and 5).

Within these SoxBac enrichments, variation in the ΔH^+^: ΔS${\text{O}}_{4}^{2-}$ ratios appeared to correspond with the presence and abundance of specific members of the *Halothiobacillus* clade (Figure 3). All enrichments showed a divergence from the expected 1:1 ratio in ΔH^+^:ΔS${\text{O}}_{4}^{2-}$ associated with complete oxidation of thiosulphate to sulfate. However, enrichments that contained higher proportions of *Halothiobacillus* sequences 10 and 14, exhibited the lowest ratios of net H^+^: S${\text{O}}_{4}^{2-}$ suggesting that relatively more sulfur disproportionation occurred in these enrichments (Warren et al., 2008). Nevertheless, the results of the enrichment experiments found circum-neutral conditions were lowered to acidic pH levels in < ~6 days in enrichments dominated by *Halothiobacillus* sp. (Table 2) which would be considered particularly fast if these rates were to ensue on an active mine site.

The identification of 16 *Halothiobacillus* genera (Figure 3) suggests this family is ecologically well adapted to the variable conditions that can occur within tailings reservoirs. The *Halothiobacillus* genera identified in this study have the capability to drive acidic conditions through thiosulphate metabolism. However, the differential net acid to sulfate generation associated with different compositional make up of *Halothiobacillus* genera within these SoxBac enrichments suggest they are doing so via different sulfur reactions. This indicates the need to more fully constrain phylogenetic diversity of this clade with respect to sulfur cycling. Further, while all *Halothiobacillus* genera identified in this study have the capability to drive acidic conditions through thiosulphate metabolism, the differential net acid to sulfate generation associated with different compositional make up of *Halothiobacillus* genera within these SoxBac enrichments suggest they are doing so via different sulfur reactions. This indicated the need to more fully constrain phylogenetic diversity of this clade with respect to sulfur cycling.

While phylogenetic resolutions were not resolvable to the species level in this study, there was a high sequence similarity of our results to two species within the same genus: *Halothiobacillus kellyi* and *Halothiobacillus neapolitanus* (Figure 4). Both *H. kellyi* and *H. neapolitanus* have been identified as mesophillic sulfur oxidizers (Kelly and Wood, 2000; Veith et al., 2012; Vikromvarasiri et al., 2015). *H. neapolitanus* has been specifically proposed as a useful sulfur oxidizer to remove hydrogen sulfide from biogas (Vikromvarasiri and Pisutpaisal, 2014) and to be capable of tolerating high NaCl concentrations (>860 mmol/L; Sievert et al., 2000) which would explain their occurrence in the high salt conditions often associated within tailing reservoirs.

Similar to *Halothiobacillus*, the closely related genus *Thiovirga* (present in Mine 1 tailing reservoir SoxBac enrichments) has also been described as a mesophilic, chemolithoautotrophic, sulfur-oxidizing bacterium (Ito et al., 2005; Yang et al., 2011). However, *Halothiobacillus or Thiovirga* from Mine 1 tailing reservoir was not successfully enriched for despite both being present in the parent waters (n.b. *Halothiobacillus* was present but at very low concentrations). The geochemical conditions of the Mine 1 tailings reservoir exhibited the lowest total sulfur concentrations (2.3 mM; Table 1), proportionally higher S${\text{O}}_{4}^{2-}$ concentrations (2.2 mM; Table 1), and thus the lowest S_react_ concentration (100 μmol L^−1^), i.e., available S substrate for sulfur oxidizers, and two orders of magnitude higher N${\text{O}}_{3}^{2-}$ (Table S1). In these waters it was the *Thiovirga* genus that was dominant from the *Halothiobacillaceae* family (Figure 3, Table S4). This result suggests that the pre-existing geochemical conditions of the tailing reservoirs (either those measured in this study or additional unknown parameters), are governing which sulfur oxidizing microbes are dominant and that which genera are present will correspondingly influence the sulfur cycling that occurs and associated acid and sulfate generation.

## Conclusions

To date, the lack of across mine comparison of both circum-neutral tailings reservoir parent and associated SoxBac enrichment microbial communities in tandem with sulfur geochemistry characterization has prevented the identification of key microbial players in these systems that could serve as indicators of important biologically catalyzed processes. While waste rock AMD has been perceived to represent tailings associated net acid generating conditions, our results here indicate that the important microbial players are likely different (Figure 5). Here, while circum-neutral MIW microbial communities were highly divergent from one another, consistent with variable MIW sample geochemistry, the SoxBac enrichments from seven of these parent MIWs converged to >70% and up to 100% abundance of *Halothiobacillus* spp. (Figure 5). The presence of *Halothiobacillus* spp. in two of the acidic parent MIWs (tailings reservoir and receiving environment samples) is consistent with the active role of this genus in catalyzing the shift to net acid generation and supporting the significance of this work beyond a culture-based study.

*Halothiobacillus* spp. diverge significantly from the well known waste rock AMD microbes *Acidithiobacillus* spp (i.e., *A. ferrooxidans* or *A. thiooxidans*, Figure 5C) underscoring the need for better characterization of microbial sulfur cycling within tailings associated mining impacted waters. The results identify a putative biological driver associated with the onset and propagation of net acid generating conditions in mine tailing reservoirs. The ability to identify these processes initiating during the early phase, would provide environmental managers with opportunities to intervene before accelerating acid generating conditions and significant environmental and economic implications ensue.

## Author Contributions

KW-M, LW, and TN contributed to experimental conception and design. KW-M and TN were responsible for field and laboratory microbial and geochemical sample preparation and analysis. GJ contributed to 16SrRNA microbial genetic sequence analysis and interpretation. JM was responsible for phylogenetic analysis. SA and CJ provided input on experimental design (specifically field collection methods for geochemical analysis) and geochemical analytics. KW-M, GJ, and LW were responsible for primary manuscript preparation incorporating input from all authors. KW-M, GJ, TN, JM, and LW all contributed to figure preparation.

### Conflict of Interest Statement

The authors declare that the research was conducted in the absence of any commercial or financial relationships that could be construed as a potential conflict of interest.
