# Supplementary material for: The Potential Role of Halothiobacillus spp. in Sulfur Oxidation and Acid Generation in Circum-Neutral Mine Tailings Reservoirs
Source: Front Microbiol. 2019 Mar 8;10:297. doi: 10.3389/fmicb.2019.00297 (PMC6418380; doi:10.3389/fmicb.2019.00297)
Supplement: Supplementary file 1 [file Data_Sheet_1.docx]

**Supplementary Material**

**The potential role of *Halothiobacillus* spp*.* in sulphur oxidation and acid generation in circumneutral mine tailings reservoirs**

Kelly Whaley-Martin^1#^, Gerdhard L. Jessen^1#&^, Tara Colenbrander Nelson^1^, Jiro F. Mori^1,%^, Simon Apte^2^, Chad Jarolimek^2^, and Lesley A. Warren*^1^

**Figure S1.** Schematic of sampling protocol for geochemistry, microbial community and SoxBac enrichments from mining impacted water (MIW) (Mine 1 to 4).

**Table S1.** *In situ* physiochemical characteristics (% saturation O_2_, conductivity, oxidation reduction potential (ORP), and nitrogen (NO_2_^-^, NO_3_^-^ ) concentrations (reported as triplicate average ± standard deviation) of tailings reservoirs sampled in 2017 for Mine 1, 2, 3 and 4, input waters (Mine 3) and receiving environment (Mine 4) that were used for SoxBac

enrichment experiments.

| Field Site | O_2_  (%) | | O_2_  mM | | Cond  (μS/cm^3^) | | ORP | | NO_2_^-^ (μmol/L) | | NO_3_^-^ (μmol/L) | |  |
| --- | --- | --- | --- | --- | --- | --- | --- | --- | --- | --- | --- | --- | --- |
| Mine 1 Tailings Pond (September) 0.5 m | | - | | - | | - | | - | | 4.8 ± 0.1 | | 245 ± 29 | |
| Mine 1 Tailings Pond (October) 0.5 m | | - | | - | | - | | - | | - | | - | |
| Mine 2 Tailings Pond (September) 0.5 m | | 87.7 | | 0.62 | | 1203 | | 168 | | 0.39 ± 0.1 | | 35 ± 7 | |
| Mine 3 Tailings Pond September 2 m | | 72.4 | | 0.26 | | 2288 | | 217 | | 3.6 ± 0.07 | | 39 ± 0.07 | |
| Mine 3 Tailings Pond (November) 0.5 m | | 30.6 | | 0.17 | | 2097 | | 111 | | 5.1 ± 0.2 | | 35 ± 4 | |
| Mine 3 Tailings Pond (November) 10 m | | 22 | | 0.15 | | 2091 | | 117 | | 5.4 ± 0.5 | | 35 ± 11 | |
| Mine 3 Input Water (November) 0.5 m | | 93.2 | | 0.75 | | 1670 | | 389 | | 0.24 ± 0.2 | | 16 ± 8 | |
| Mine 4 Tailings Pond (September) 7 m | | 88.6 | | 0.60 | | 2714 | | 113 | | 18 ± 0.1 | | 54 ± 14 | |
| Mine 4 Receiving Environment (September) 0.5 m | | 39.1 | | 0.27 | | 2322 | | 308 | | 2.8 ± 0.1 | | 78 ± 8 | |

**Neutrophilic Sulphur Oxidizing Bacterial (SoxBac) Enrichment Media**

Sulphur oxidizing bacterial enrichment media was prepared with the following procedure in 1 L batches. Solution “T” was prepared by dissolving 50 g of EDTA disodium salt into 400 mL of water followed by addition of 9 g of NaOH into the EDTA solution. The following salts were added individually to 30 mL of water and added to the EDTA solution: 5 g of ZnSO_4_•7H_2_O, 5 g of CaCl_2_ (or 7.34 g of CaCl_2_•2H_2_O), 2.5 g of MnCl_2_•6H_2_O, 0.5 g of CoCl_2_•6H_2_O, 0.5 g of ammonium molybdate, 5 g of FeSO_4_•7H_2_O and 0.2 g of CuSO_4_•5H_2_O. “Solution 1” was prepared with 90 mL of 1.1% (w/v) K_2_HPO_4_ was added to 400 mL of tap water. “Solution 2” was prepared with 5 g of Na_2_S_2_O_3_, 90 mL of 0.44% (w/v) NH_4_Cl, 90 mL of 0.11% (w/v) MgSO_4_, 2.2 mL of Solution T and 320 mL of tap water. Solution 1 and 2 were sterilized separately by filtration (<0.2µm) or autoclaving and combined.

**
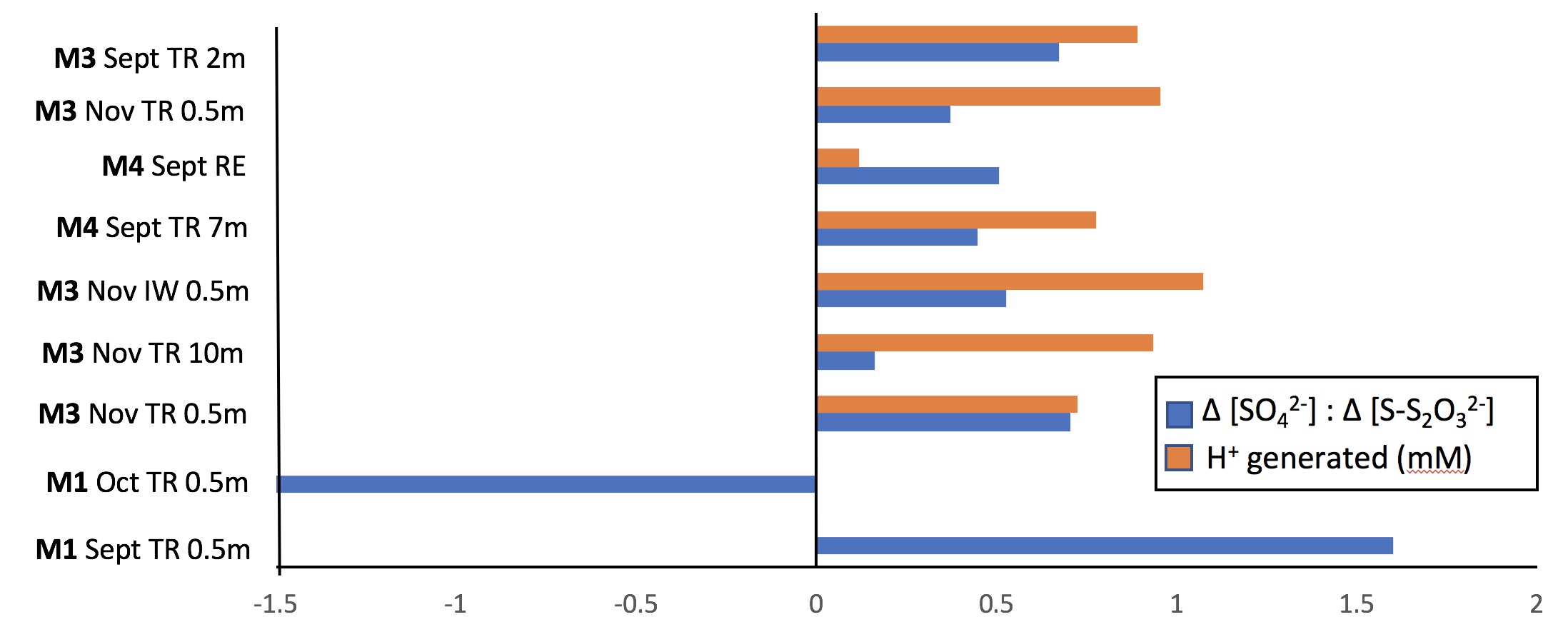
**

**Figure S2.** The net concentration change of sulphate to thiosulfate (∆[SO_4_^2-^]:∆[S-S_2_O_3_^2-^]) and the net production of [H^+^] from t = initial vs t = final for each of the nine MIW sample SoxBac enrichments.

|  | **Number of reads** | **Unique seq** | | **Richness** | **1/D** | **Evenness** |
| --- | --- | --- | --- | --- | --- | --- |
| **M1 Sept TR 0.5m** | 117,274 | 62 | 163 | | 6.1 | 0.5 |
| **M1 Oct TR 0.5m** | 82,933 | 35 | 118 | | 19.2 | 0.7 |
| **M2 Sept TR 0.5m** | 121,553 | 349 | 492 | | 34.5 | 0.7 |
| **M3 Sept TR 10m** | 108,581 | 24 | 97 | | 1.9 | 0.3 |
| **M3 Nov TR 0.5m** | 108,627 | 43 | 148 | | 4.2 | 0.4 |
| **M3 Nov TR 10m** | 129,863 | 66 | 184 | | 4.0 | 0.4 |
| **M3 Nov IW 0.5m** | 124,640 | 314 | 408 | | 3.8 | 0.4 |
| **M4 Sept TR 7m** | 112,227 | 229 | 249 | | 9.5 | 0.6 |
| **M4 Sept RE** | 89,465 | 578 | 702 | | 8.5 | 0.5 |
| **E M1 Sept TR 0.5m** | 169,205 | 7 | 20 | | 3.4 | 0.5 |
| **E M1 Oct TR 0.5m** | 82,227 | 6 | 21 | | 1.5 | 0.2 |
| **E M2 Sept TR 0.5m** | 202,652 | 3 | 22 | | 1.6 | 0.2 |
| **E M3 Sept TR 2m** | 177,701 | 26 | 63 | | 2.0 | 0.2 |
| **E M3 Nov TR 0.5m** | 135,352 | 5 | 28 | | 1.1 | 0.1 |
| **E M3 Nov TR 10m** | 149,566 | 21 | 40 | | 1.5 | 0.2 |
| **E M3 Nov IW 0.5m** | 108,711 | 23 | 28 | | 2.8 | 0.4 |
| **E M4 Sept TR 7m** | 184,230 | 59 | 139 | | 1.4 | 0.1 |
| **E M4 Sept RE** | 168,471 | 11 | 39 | | 2.6 | 0.4 |

**Table S2.** Number of unique sequences, richness, reciprocal of Simpson index (1/D) and evenness between mines, enrichments and mine environments (based on 16S rRNA gene amplicons).

**Table S3.** Percentage of shared sequences and β-diversity between mines, enrichments and mine environments (based on 16S rRNA gene amplicons). The lower triangle is the percentage of shared sequences, while the upper triangle is β-diversity (calculated as Bray-Curtis dissimilarity; 0, completely similar; 1 completely different. M, mine; E, enrichment; TR, tailings reservoir; IW, input water; RE, receiving environment. Sampling depth is surface water unless depicted (m).

|  | **M1 Sept TR 0.5** | **E M1 Sept TR 0.5** | **M1 Oct TR 0.5** | **E M1 Oct TR 0.5** | **M2 Sept TR 0.5** | **E M2 Sept TR 0.5** | **M3 Sept TR 10m** | **E M3 Sept TR 2m** | | **M3 Nov TR 0.5** | **E M3 Nov TR 0.5** | **M3 Nov TR 10** | **E M3 Nov TR 10** | **M3 Nov IW 0.5** | **E M3 Nov IW 0.5** | **M4 Sept TR 7** | **E M4 Sept TR 7** | **M4 Sept RE** | **E M4 Sept RE** |
| --- | --- | --- | --- | --- | --- | --- | --- | --- | --- | --- | --- | --- | --- | --- | --- | --- | --- | --- | --- |
| **M1 Sept TR 0.5m** |  | 1 | 0.66 | 1 | 0.96 | 1 | 1 | 1 | | 1 | 0.66 | 1 | 0.96 | 1 | 1 | 1 | 1 | 0.66 | 1 |
| **E M1 Sept TR 0.5m** | 5 |  | 0.99 | 0.54 | 1 | 1 | 0.97 | 1 | | 1 | 0.99 | 1 | 0.82 | 1 | 0.9 | 1 | 1 | 1 | 1 |
| **M1 Oct TR 0.5m** | 34 | 4 |  | 1 | 0.93 | 1 | 0.99 | 1 | | 1 | 1 | 1 | 1 | 1 | 1 | 0.87 | 1 | 1 | 1 |
| **E M1 Oct TR 0.5m** | 3 | 24 | 4 |  | 1 | 1 | 0.97 | 1 | 1 | | 0.99 | 1 | 0.82 | 1 | 0.9 | 1 | 1 | 1 | 1 |
| **M2 Sept TR 0.5m** | 8 | 1 | 5 | 1 |  | 1 | 0.99 | 1 | | 1 | 1 | 0.99 | 1 | 1 | 1 | 0.88 | 0.99 | 0.99 | 1 |
| **E M2 Sept TR 0.5m** | 5 | 11 | 4 | 8 | 2 |  | 0.27 | 0.54 | | 0.84 | 0.22 | 0.88 | 0.23 | 1 | 0.34 | 0.97 | 0.08 | 0.72 | 0.41 |
| **M3 Sept TR 10m** | 5 | 5 | 4 | 7 | 4 | 7 |  | 0.77 | | 0.76 | 0.26 | 0.8 | 0.25 | 0.99 | 0.54 | 0.96 | 0.27 | 0.72 | 0.41 |
| **E M3 Sept TR 2m** | 3 | 8 | 3 | 9 | 1 | 13 | 12 |  | | 0.84 | 0.76 | 0.88 | 0.76 | 1 | 0.37 | 0.97 | 0.61 | 0.76 | 0.75 |
| **M3 Nov TR 0.5m** | 3 | 3 | 3 | 4 | 3 | 5 | 23 | 10 | |  | 0.84 | 0.06 | 0.84 | 0.98 | 0.84 | 0.96 | 0.84 | 0.83 | 0.84 |
| **E M3 Nov TR 0.5m** | 6 | 14 | 4 | 17 | 1 | 14 | 7 | 11 | | 4 |  | 0.88 | 0.17 | 1 | 0.54 | 0.97 | 0.17 | 0.72 | 0.4 |
| **M3 Nov TR 10m** | 3 | 3 | 3 | 3 | 4 | 5 | 24 | 9 | | 37 | 3 |  | 0.88 | 0.98 | 0.88 | 0.96 | 0.88 | 0.87 | 0.88 |
| **E M3 Nov TR 10m** | 3 | 9 | 2 | 13 | 0 | 7 | 5 | 6 | | 3 | 24 | 3 |  | 1 | 0.5 | 0.97 | 0.19 | 0.73 | 0.41 |
| **M3 Nov IW 0.5m** | 2 | 1 | 2 | 1 | 2 | 2 | 5 | 4 | | 7 | 1 | 8 | 1 |  | 1 | 1 | 1 | 0.99 | 1 |
| **E M3 Nov IW 0.5m** | 4 | 9 | 3 | 9 | 1 | 9 | 4 | 8 | | 3 | 24 | 2 | 15 | 1 |  | 0.97 | 0.41 | 0.69 | 0.56 |
| **M4 Sept TR 7m** | 6 | 2 | 8 | 3 | 5 | 3 | 7 | 5 | | 7 | 3 | 9 | 2 | 4 | 2 |  | 0.97 | 0.95 | 0.96 |
| **E M4 Sept TR 7m** | 6 | 3 | 5 | 4 | 10 | 3 | 3 | 5 | | 4 | 6 | 3 | 6 | 1 | 6 | 5 |  | 0.72 | 0.41 |
| **M4 Sept RE** | 2 | 1 | 1 | 1 | 3 | 1 | 3 | 3 | | 4 | 1 | 4 | 1 | 5 | 1 | 9 | 2 |  | 0.58 |
| **E M4 Sept RE** | 3 | 4 | 1 | 3 | 2 | 17 | 5 | 13 | | 6 | 16 | 5 | 8 | 2 | 14 | 2 | 7 | 2 |  |

**Table S4.** Relative abundance (%) of all the single absolute sequences clustered at Genus level for the Order *Halothiobacillaceae* (obtained by Illumina sequencing of 16S rRNA gene amplicons) for the different mines, mining environments and its enrichments. Relative abundance is calculated per sample and relative to the whole microbial community

|  | Halothiobacillus1 | Halothiobacillus2 | Halothiobacillus3 | Halothiobacillus4 | Halothiobacillus5 | Halothiobacillus6 | Halothiobacillus7 | Halothiobacillus8 | Halothiobacillus9 | Halothiobacillus10 | Halothiobacillus11 | Halothiobacillus12 | Halothiobacillus13 | Halothiobacillus14 | Halothiobacillus15 | Halothiobacillus16 | Thiovirga1 | Thiovirga2 | Thiovirga3 | Thiovirga4 | unclassified_Halothiobacilliceae1 | unclassified_Halothiobacilliceae2 | unclassified_Halothiobacilliceae3 | unclassified_Halothiobacilliceae4 | unclassified_Halothiobacilliceae5 |
| --- | --- | --- | --- | --- | --- | --- | --- | --- | --- | --- | --- | --- | --- | --- | --- | --- | --- | --- | --- | --- | --- | --- | --- | --- | --- |
| M1 Oct TR 0.5m |  |  |  |  | 0.03 |  |  |  |  | <0.01 |  |  |  |  |  |  |  |  |  | 10.65 |  |  |  |  |  |
| M4 Sept TR 7m |  |  |  |  | 3.00 |  |  |  |  | 0.08 |  |  |  | 0.15 |  | 0.21 |  | 0.19 |  | 5.42 | 0.12 | 0.90 |  | 0.39 | 0.02 |
| M1 Sept TR 0.5m |  |  |  |  | 0.05 |  |  |  |  | <0.01 |  |  |  |  |  |  |  |  |  | 1.81 |  |  |  |  |  |
| M2 Sept TR 0.5m |  |  |  |  | 0.11 |  |  |  |  |  |  |  |  | <0.01 |  | <0.01 | 0.07 |  | 0.10 | 0.06 |  | 3.77 | 0.96 | 0.08 |  |
| M3 Nov IW 0.5m |  |  |  |  | 0.04 |  |  |  |  | 0.02 |  |  |  |  |  |  |  |  |  | 0.07 |  |  |  |  |  |
| E M1 Sept TR 0.5m |  |  |  |  | 0.02 |  |  |  |  |  |  |  |  |  |  |  |  |  |  | 0.05 |  |  |  |  |  |
| E M1 Oct TR 0.5m |  |  |  |  | 0.01 |  |  |  |  | 0.02 |  |  |  |  |  |  |  |  |  | <0.01 |  |  |  |  |  |
| E M3 Nov TR 0.5m | 0.05 |  |  |  | 96.23 | 0.02 |  |  |  | 0.76 |  |  |  |  | <0.01 | 0.04 |  |  |  | <0.01 |  |  |  |  |  |
| E M3 Nov TR 10m |  | 0.05 |  |  | 81.07 | 0.01 |  | 0.02 |  | 0.21 |  |  |  |  |  |  |  |  |  |  |  |  |  |  |  |
| M3 TR Sept 10m |  |  | 5.21 |  | 72.52 |  | 0.01 |  | 0.23 | 0.01 |  |  |  |  |  |  |  |  |  | 0.20 |  |  |  |  |  |
| E M2 Sept TR 0.5m |  |  |  |  | 76.85 |  | 0.02 |  |  | 22.65 |  |  |  |  |  | 0.31 |  | <0.01 |  |  |  | <0.01 |  |  |  |
| E M4 Sept TR 7m |  |  |  | 0.17 | 81.98 | 0.02 |  |  |  | 15.59 |  |  |  | 0.01 |  |  | <0.01 |  |  | <0.01 |  |  | <0.01 |  |  |
| E M4 Sept RE |  |  |  |  | 58.78 |  | 0.02 |  |  | 0.10 |  |  |  | 14.77 |  | 3.16 |  |  |  |  |  |  |  |  |  |
| E M3 Nov IW 0.5m |  |  |  |  | 43.43 |  |  |  |  | 39.67 | 0.01 |  | 0.13 | 0.14 |  |  |  |  |  |  |  |  |  |  |  |
| E M3 Sept TR 2m |  |  | 0.11 |  | 23.24 |  |  |  |  | 66.85 |  | <0.01 |  | 0.05 |  | 0.18 |  |  |  | <0.01 |  |  |  |  |  |
| M3 Nov TR 0.5m |  |  |  |  | 15.96 |  |  |  |  | 0.01 |  |  |  |  |  |  |  |  |  | 0.08 |  |  |  |  |  |
| M3 Nov TR 10m |  |  |  |  | 11.59 |  |  |  | 0.08 | 0.01 |  |  |  |  |  |  |  |  |  | 0.08 |  |  |  |  |  |
| M4 Sept RE |  |  | 0.56 |  | 27.18 |  |  |  | 8.23 | 0.01 |  |  |  | 10.40 |  | 5.72 |  | 0.07 |  | 0.04 | 0.09 | 0.13 |  | 0.06 | 0.04 |
